# Posterior-Contact Soft-ZUPT for Foot-Mounted Inertial Navigation: Uncertainty-Aware Pseudo-Observation Modeling

**DOI:** 10.3390/s26103033

**Published:** 2026-05-11

**Authors:** Yanbiao Gao, Zhongliang Deng

**Affiliations:** School of Electronic Engineering, Beijing University of Posts and Telecommunications, Beijing 100876, China; yanbiao.gao@bupt.edu.cn

**Keywords:** foot-mounted inertial navigation, zero-velocity update, soft ZUPT, probabilistic pseudo-observation modeling, error-state Kalman filter

## Abstract

Foot-mounted inertial navigation is an important option for pedestrian positioning when external localization infrastructure is limited, unavailable, or unreliable. Its performance, however, depends heavily on zero-velocity updates (ZUPTs), which can become overconfident during heel-strike, toe-off, and other non-ideal contact phases. This paper formulates ZUPT as probabilistic pseudo-observation modeling and introduces posterior-contact soft-ZUPT, in which update strength is controlled by a detector-derived contact prior and an innovation-conditioned posterior within a single error-state Kalman filter. Experiments on the 56-trial foot-mounted VICON benchmark under a two-fold development/evaluation protocol compare the method against hard-ZUPT, robust soft-ZUPT, contact soft-ZUPT, and a foot-instability-based adaptive covariance (FIBA)-like baseline. Posterior-contact soft-ZUPT improves difficult-trial robustness and upper-tail error suppression while remaining competitive in overall accuracy, achieving the best selected p95 and tail-risk values among the compared methods. These results indicate that probabilistic reliability modeling can soften ZUPT effectively while preserving a lightweight single-filter implementation.

## 1. Introduction

Reliable pedestrian positioning is important in a wide range of applications, including indoor location-based services, industrial inspection, emergency response, public-safety operations, and navigation in underground or structurally complex environments [1,2,3]. In many such settings, localization cannot always depend on external infrastructure such as satellite signals, wireless networks, beacons, or pre-mapped environments. Signal blockage, infrastructure unavailability, deployment cost, and environmental change all motivate positioning methods that remain functional when external support is limited, unreliable, or absent [1,2].

In these infrastructure-unavailable scenarios, inertial navigation is attractive because it is self-contained, continuous, and does not require pre-installed anchors or online connectivity [4,5]. For pedestrian navigation in particular, foot-mounted inertial navigation has become one of the most important infrastructure-independent paradigms because foot motion exhibits a strong gait structure with recurring support phases [6,7]. That structure makes it possible to exploit near-zero-velocity intervals during stance as natural correction opportunities, which is the central reason why foot-mounted systems remain effective despite the drift of low-cost microelectromechanical inertial sensors [6,8,9].

The standard correction mechanism is the zero-velocity update (ZUPT). By injecting zero-velocity pseudo-observations during stance, ZUPT-aided foot-mounted navigation can sharply reduce accumulated velocity error and long-horizon position drift [8,9,10]. Its effectiveness, however, depends critically on whether the update is applied at the right time and with the right confidence. In realistic walking data, heel-strike, toe-off, transient contact, and other non-ideal support phases make the zero-velocity hypothesis uncertain, and several studies have shown that these phases can violate the assumptions behind idealized stance detection and fixed-strength updates [11,12,13]. More fundamentally, prior analyses have pointed out that the ZUPT pseudo-observation itself is often affected by non-negligible modeling error rather than only by detector error [14]. As a result, a detector-triggered fixed-covariance update can become overconfident in difficult intervals. In this case, the filter gives excessive weight to an imperfect zero-velocity pseudo-observation and may force a large correction to the velocity state and to the coupled position and attitude states, which can in turn produce large trajectory errors.

This limitation has motivated two broad lines of research. One line improves zero-velocity detection itself through adaptive thresholds, interval extraction, and learning-based detectors. Another instead softens the pseudo-observation itself through continuous covariance modulation or explicit latent-state inference [6,10,15,16]. Together, these studies define the relevant design space, but they also leave a practical gap. Detector-centric methods improve when a ZUPT candidate should be formed, but they usually still pass a binary or thresholded stance output to the filter and therefore do not specify how strongly an uncertain zero-velocity pseudo-observation should be trusted. Fully probabilistic standstill models can represent this uncertainty more explicitly, but they often rely on switching-state or mode-conditioned inference. What remains missing is therefore a lightweight single-filter update rule that uses both detector-derived contact confidence and innovation consistency to set the strength of each ZUPT correction.

This paper addresses that gap through posterior-contact soft-ZUPT. The main idea is to treat ZUPT as a stochastic pseudo-observation whose reliability depends on both contact confidence and the current innovation. Starting from a detector-derived contact prior, the method applies lightweight temporal smoothing and then updates the contact belief using the innovation likelihood. Intuitively, a candidate ZUPT is trusted when the detector indicates contact and the measured innovation remains consistent with near-zero velocity; when the innovation is inconsistent, the posterior-contact belief decreases and the effective measurement covariance is inflated. This posterior-contact belief is expressed within a single error-state Kalman filter (ESKF) through a posterior-weighted effective covariance, yielding a practical approximation to latent contact-state inference without departing from the standard correction framework.

The main contributions of this paper are as follows:

- We formulate soft-ZUPT as probabilistic pseudo-observation modeling, in which each zero-velocity pseudo-observation is assigned an uncertainty-aware reliability. This formulation allows detector-derived contact confidence and innovation consistency to jointly modulate the ZUPT correction strength within a single ESKF.
- We develop a lightweight posterior-contact realization that combines detector-derived contact confidence, limited temporal persistence, and innovation-conditioned posterior correction while preserving the computational structure of a standard foot-mounted inertial navigation system.
- We evaluate this formulation on the foot-mounted VICON benchmark against hard-ZUPT, robust soft-ZUPT, contact soft-ZUPT, and a foot-instability-based adaptive covariance (FIBA)-like baseline. The results show that posterior-contact soft-ZUPT provides the strongest upper-tail error suppression and difficult-trial mitigation among the compared methods while remaining competitive in overall accuracy.

The remainder of this paper is organized as follows. Section 2 reviews the most relevant literature on hard- and soft-ZUPT, detector-centric adaptation, adaptive-covariance baselines, and probabilistic standstill modeling. Section 3 presents the posterior-contact formulation and its single-ESKF realization. Section 4 describes the benchmark, protocol, compared methods, and evaluation metrics. Section 5 reports the empirical results. Section 6 discusses the main findings and methodological implications, and Section 7 concludes the paper.

## 2. Related Work

### 2.1. Classical Detector-Triggered ZUPT

Classical foot-mounted inertial navigation relies on a detector-triggered correction pipeline: a zero-velocity detector decides whether the foot is in stance, and a fixed-covariance pseudo-observation is applied once the detector fires [8,9]. This structure is simple, computationally efficient, and highly effective when the stance intervals are cleanly separated from swing motion. It therefore remains the standard point of departure for most foot-mounted inertial navigation systems [6,10].

At the same time, this pipeline treats the zero-velocity hypothesis as a binary event. Once the detector triggers, the update is typically applied with a fixed information strength, without explicitly representing uncertainty in the contact process or in the local consistency of the innovation. Prior work has also emphasized that the ZUPT pseudo-observation itself is subject to non-negligible modeling error rather than only detector error [14]. For a ZUPT pseudo-observation, modeling error means that the foot may still have residual motion during a detected support interval, for example because of heel-strike, toe-off, rolling contact, foot slip, or sensor attachment motion; the zero-velocity pseudo-observation is therefore an approximation whose reliability can vary across contact conditions. These limitations motivate uncertainty-aware update modeling, where the filter update is allowed to vary in strength rather than treating every detected stance interval as an equally reliable constraint. Similar uncertainty-aware ideas appear in adaptive-covariance and probabilistic standstill formulations [15,16].

### 2.2. Detector-Centric Adaptation

A large body of work improves ZUPT robustness primarily through better detector design. Bayesian threshold adaptation showed early on that a fixed detector threshold can be too brittle across changing motion regimes [17]. That detector-centric line has since expanded through adaptive interval extraction, threshold-free phase segmentation, and learning-based zero-velocity classification from inertial data, including long short-term memory-based detection and more robust learned detectors that combine motion classification, adaptive thresholding, and augmentation across motion types and sensors [13,18,19,20,21]. Closely related detector-adjacent work continues the same theme through weighted multi-gait interval extraction, impact-aware foot-motion reconstruction for ramps and stairs, and terrain-conditioned parameter adaptation [22,23,24].

These studies strengthen the detector side of the pipeline, but most still end at a detector decision or detector score rather than a probabilistic update model. In that sense, they clarify how stance evidence should be extracted and adapted, but they do not by themselves resolve how detector-derived uncertainty should modulate the pseudo-observation once a ZUPT candidate is formed. This modulation is important for performance because an overconfident update during an uncertain contact interval can inject a harmful correction, whereas an uncertainty-aware update model can reduce the update strength when the zero-velocity evidence is weak or inconsistent, thereby reducing the risk of large-error failures and upper-tail error growth.

### 2.3. Update-Centric Covariance Adaptation

A neighboring line of work shifts attention from detector design to update design. Instead of relying on a binary stance trigger followed by a fixed-strength ZUPT, these methods adjust the pseudo-observation strength itself through adaptive weighting or covariance modulation. This broader idea appears in adaptive weight-updating strategies for foot-mounted pedestrian navigation and in approaches that refine the update behavior by adapting the inferred zero-velocity interval or the contribution of the correction step [25,26,27].

A representative example in this direction is the FIBA covariance approach of Jao and Shkel, which continuously maps a foot-instability statistic into the ZUPT covariance and thereby bypasses a binary stance detector altogether [15]. Their results showed improved positioning accuracy across multiple pedestrian motion conditions and reduced reliance on a manually selected stance-detection threshold, making FIBA a strong update-centric reference method. Together, these methods define an update-centric alternative to detector-triggered ZUPT by retaining the standard recursive filter structure while replacing binary activation with adaptive weighting or continuous covariance modulation.

### 2.4. Probabilistic Standstill Modeling

Another closely related line of work models standstill or contact as a latent state and uses posterior inference to determine update behavior. Early hidden Markov model-style formulations already treated standstill detection probabilistically in foot-mounted inertial measurement unit (IMU) systems [28,29]. More recent work has pushed this idea further by coupling motion classification and state estimation through jump Markov models, mode-conditioned filtering, or filter-bank realizations [16,30]. These methods provide a principled probabilistic treatment of standstill uncertainty and make explicit the fact that the zero-velocity hypothesis need not be represented as a purely hard decision.

These methods establish the value of probabilistic contact reasoning, but their explicit switching-state realizations remain heavier to implement and integrate than a standard single-filter ZUPT pipeline. A lighter probabilistic update formulation within a conventional recursive estimator remains less explored.

## 3. Methodology

### 3.1. System Model

Foot-mounted inertial navigation can be formulated as a recursive state-estimation problem. At each time step, IMU specific-force and angular-rate measurements drive the nominal strapdown propagation, while an error-state Kalman filter (ESKF) accounts for local linearized uncertainty around that trajectory. Let ${\bar{x}}_{k}$ denote the nominal state and $\delta x_{k}$ the associated error state. The propagation step is written abstractly as

(1) $${\bar{x}}_{k}=f\left({\bar{x}}_{k-1},u_{k}\right),\;\delta x_{k}=F_{k-1}\delta x_{k-1}+G_{k-1}n_{k-1},$$

with process noise $n_{k-1}∼N\left(0,Q\right)$ and covariance recursion

(2) $$P_{k}^{-}=F_{k-1}P_{k-1}^{+}F_{k-1}^{⊤}+G_{k-1}QG_{k-1}^{⊤}.$$

Within this standard foot-mounted ESKF framework, drift is controlled through intermittent zero-velocity updates (ZUPTs), which act as corrective pseudo-observations during suitable phases of the gait cycle. The methodology developed below preserves this propagation framework and augments the correction step with a probabilistic reliability model for ZUPT.

### 3.2. Zero-Velocity Pseudo-Observation Model

In the standard correction step, the navigation error state at time step *k* is written as

(3) $$x_{k}={\left[p_{k}^{⊤},\;v_{k}^{⊤},\;\theta_{k}^{⊤}\right]}^{⊤},$$

where $p_{k}$, $v_{k}$, and $\theta_{k}$ denote position, velocity, and attitude error states, respectively. A classical hard-ZUPT assumes that, once a stance detector declares the foot to be stationary, a zero-velocity pseudo-observation is imposed with fixed confidence:

(4) $$z_{k}=0=Hx_{k}+\nu_{k},$$

where *H* selects the velocity components and $\nu_{k}∼N\left(0,R_{0}\right)$. Under this formulation, a detector determines whether the zero-velocity hypothesis is activated, and the filter then applies the corresponding pseudo-observation with covariance $R_{0}$. This hard-ZUPT construction is effective when foot contact is clean and stable, but it treats the reliability of the zero-velocity hypothesis as fixed once the update is triggered. In practice, that reliability varies substantially across time, especially near ambiguous or transitional contact intervals.

### 3.3. Latent Contact Observation Model

To represent this time-varying reliability within the same pseudo-observation structure, we introduce a latent contact variable $s_{k}\in \left\{0,1\right\}$. Conditioned on $s_{k}$, the zero-velocity pseudo-observation is retained, but its covariance depends on the contact mode. This dependence can be written through a mode-dependent covariance scale $c(s_{k})$:

(5) $$z_{k}=0=Hx_{k}+\nu_{k}^{\left(s_{k}\right)},\;\nu_{k}^{\left(s_{k}\right)}∼N\;\left(0,c\left(s_{k}\right)R_{0}\right),\;c\left(s_{k}\right)=\left\{\begin{array}{cc} 1, & s_{k}=1,\\ c, & s_{k}=0, \end{array}\right.$$

where c>1 is a covariance inflation factor for the non-contact mode. The limiting case c→+∞ corresponds to removing the zero-velocity information in the non-contact mode; in the implemented single-filter approximation, we use a large but finite *c* so that the non-contact mode contributes very small precision while keeping the update numerically well conditioned. Under this model, $s_{k}=1$ corresponds to a high-confidence zero-velocity correction, whereas $s_{k}=0$ corresponds to a weakly trusted pseudo-observation with inflated covariance. The latent contact variable therefore indexes the reliability mode of the ZUPT correction at time step *k*, while preserving the same underlying observation structure.

### 3.4. Detector-Derived Contact Prior

Classical detector-based ZUPT pipelines begin by computing a short-window test statistic that measures how compatible the current inertial measurements are with the zero-velocity hypothesis. In this work, that evidence is represented by a SHOE (stance hypothesis optimal estimation) statistic [9,10], denoted by $T_{k}$, and computed over a local window $W_{k}$ of *W* samples as

(6) $$T_{k}=\frac{1}{W}\sum_{n\in W_{k}}\left[\frac{1}{\sigma_{a}^{2}}{∥a_{n}-g\frac{{\bar{a}}_{k}}{∥{\bar{a}}_{k}∥}∥}^{2}+\frac{1}{\sigma_{\omega }^{2}}{∥\omega_{n}∥}^{2}\right],$$

where $a_{n}$ and $\omega_{n}$ denote the accelerometer and gyroscope measurements, respectively, ${\bar{a}}_{k}$ is the mean accelerometer measurement over the window, and $\sigma_{a}^{2}$ and $\sigma_{\omega }^{2}$ are the corresponding detector normalization constants. The first term measures deviation from gravity-aligned acceleration, while the second term measures angular-rate energy. Smaller values of $T_{k}$ therefore indicate greater compatibility with the zero-velocity hypothesis. Let *G* denote the corresponding detector threshold. Thus, $T_{k}$ is computed first from the inertial measurements as the detector statistic, while $q_{k}$ is the derived soft contact-confidence score used by the proposed update model.

In the present formulation, we map the computed detector statistic to a soft contact score:

(7) $$q_{k}=\sigma \;\left(\alpha \log_{10}\frac{G}{T_{k}}\right),$$

where σ(·) is the sigmoid function and α controls the transition sharpness. Under this mapping, detector values below the threshold produce larger contact confidence, whereas values above the threshold reduce that confidence.

To encode temporal persistence in the contact state, the previous posterior is propagated through a symmetric two-state Markov prior:

(8) $${\tilde{\pi }}_{k}^{-}=p_{\mathrm{stay}}\pi_{k-1}^{+}+\left(1-p_{\mathrm{stay}}\right)\left(1-\pi_{k-1}^{+}\right),$$

where $p_{\mathrm{stay}}\in \left[0.5,1\right)$ is the stay probability. When $p_{\mathrm{stay}}=0.5$, this prior becomes non-committal, whereas larger values impose stronger temporal persistence. The detector-derived score $q_{k}$ is then fused with this propagated prior through a Bernoulli update:

(9) $$\pi_{k}^{-}=\frac{q_{k}{\tilde{\pi }}_{k}^{-}}{q_{k}{\tilde{\pi }}_{k}^{-}+\left(1-q_{k}\right)\left(1-{\tilde{\pi }}_{k}^{-}\right)}.$$

The resulting quantity $\pi_{k}^{-}$ is therefore the prior contact probability before the zero-velocity innovation is evaluated. In the experiments, the SHOE statistic was computed with a fixed local window of W=5 samples (25 ms at 200 Hz) for all compared methods. Candidate ZUPT update samples are then generated by applying the selected minimum contact-probability gate $\pi_{\min}$ to the temporally smoothed detector-derived prior; consecutive active samples form the update intervals, and the posterior-contact rule subsequently modulates their correction strength.

### 3.5. Innovation-Conditioned Posterior Contact

Given the prior contact probability $\pi_{k}^{-}$, we use the innovation associated with the zero-velocity hypothesis to update the contact belief:

(10) $$r_{k}=-H{\hat{x}}_{k}^{-}.$$

This innovation is evaluated under the two contact-dependent pseudo-observation modes introduced above. The contact mode uses covariance $R_{0}$, whereas the non-contact mode uses the inflated covariance $cR_{0}$ with c>1. Their likelihoods are written as

(11) $$\ell_{k}^{\left(1\right)}=N\left(r_{k};0,S_{k}^{\left(1\right)}\right),\;\ell_{k}^{\left(0\right)}=N\left(r_{k};0,S_{k}^{\left(0\right)}\right),$$

where

(12) $$S_{k}^{\left(1\right)}=HP_{k}^{-}H^{⊤}+R_{0},\;S_{k}^{\left(0\right)}=HP_{k}^{-}H^{⊤}+cR_{0}.$$

Combining the prior contact belief with these innovation likelihoods yields the posterior-contact probability

(13) $$\pi_{k}^{+}=\frac{\pi_{k}^{-}\ell_{k}^{\left(1\right)}}{\pi_{k}^{-}\ell_{k}^{\left(1\right)}+\left(1-\pi_{k}^{-}\right)\ell_{k}^{\left(0\right)}}.$$

Equivalently, the update can be written in log-odds form as

(14) $$\log\frac{\pi_{k}^{+}}{1-\pi_{k}^{+}}=\log\frac{\pi_{k}^{-}}{1-\pi_{k}^{-}}+\log\frac{\ell_{k}^{\left(1\right)}}{\ell_{k}^{\left(0\right)}}.$$

Under this formulation, the detector-derived prior contributes the initial contact odds, while the innovation contributes a likelihood ratio that revises those odds at the current time step. The resulting quantity $\pi_{k}^{+}$ is therefore the posterior-contact probability after incorporating both detector evidence and innovation consistency.

### 3.6. Posterior-Contact Update Rule

The posterior-contact probability $\pi_{k}^{+}$ determines how strongly the zero-velocity pseudo-observation should constrain the filter at time step *k*. Under the two-mode contact model, the contact mode contributes covariance $R_{0}$, whereas the non-contact mode contributes the inflated covariance $cR_{0}$. The corresponding correction is a posterior mixture over the two mode-conditioned pseudo-observation models.

To obtain a practical update rule within the standard ESKF correction framework, we collapse this posterior mixture into a single Gaussian pseudo-observation by matching posterior expected observation precision. Let

(15) $$\lambda_{k}=\pi_{k}^{+}+\frac{1-\pi_{k}^{+}}{c}.$$

Because $\pi_{k}^{+}\in \left[0,1\right]$ and c>1, the precision scale satisfies $\lambda_{k}\in \left[1/c,1\right]$. Thus, $\lambda_{k}$ equals 1 for a high-confidence contact update and approaches 1/c for a low-confidence update. This yields the effective covariance

(16) $$R_{k}^{\mathrm{eff}}=\frac{R_{0}}{\lambda_{k}}.$$

The zero-velocity correction is then carried out using the standard ESKF update with $R_{k}^{\mathrm{eff}}$ in place of the fixed hard-ZUPT covariance.

**Proposition 1.**  *Assume the mode-conditioned pseudo-observation precisions are $R_{0}^{-1}$ in contact and ${\left(cR_{0}\right)}^{-1}$ in non-contact, and approximate their posterior mixture by a single Gaussian pseudo-observation whose precision equals the posterior expected precision. Then the corresponding effective covariance is*

(17) $$R_{k}^{\mathrm{eff}}=\frac{R_{0}}{\lambda_{k}},\;\lambda_{k}=\pi_{k}^{+}+\frac{1-\pi_{k}^{+}}{c}.$$

**Proof.**  Conditioned on the innovation, the posterior expected precision is

(18) $$E\;\left[\Lambda_{k}|r_{k}\right]=\pi_{k}^{+}R_{0}^{-1}+\left(1-\pi_{k}^{+}\right){\left(cR_{0}\right)}^{-1}=\left(\pi_{k}^{+}+\frac{1-\pi_{k}^{+}}{c}\right)R_{0}^{-1}=\lambda_{k}R_{0}^{-1}.$$

Matching this expected precision with a single Gaussian pseudo-observation gives ${\left(R_{k}^{\mathrm{eff}}\right)}^{-1}=\lambda_{k}R_{0}^{-1}$, and therefore $R_{k}^{\mathrm{eff}}=R_{0}/\lambda_{k}$.    □

When $\pi_{k}^{+}\to 1$, the effective covariance reduces to $R_{0}$ and the method recovers hard-ZUPT. When $\pi_{k}^{+}\to 0$, the covariance approaches $cR_{0}$, so the correction is weakened toward the designated low-confidence mode. For intermediate posterior values, the effective covariance varies continuously between these two endpoints. The posterior-contact probability is used here as an update-control variable that continuously adjusts the ZUPT correction strength according to detector-derived contact evidence and innovation consistency, thereby reducing overconfident corrections during ambiguous contact intervals. The resulting posterior-contact update is summarized in Algorithm 1.
**Algorithm 1** Posterior-contact soft-ZUPT update at time step *k***Require:** $\pi_{k-1}^{+}$, ${\hat{x}}_{k}^{-}$, $W_{k}$, $R_{0}$, *c***Ensure:** $\pi_{k}^{+}$, $R_{k}^{\mathrm{eff}}$1:Compute the detector statistic $T_{k}$ and the soft contact score $q_{k}$;2:Propagate the previous posterior through the Markov persistence prior to obtain ${\tilde{\pi }}_{k}^{-}$, and fuse it with $q_{k}$ to obtain the contact prior $\pi_{k}^{-}$;3:Compute the zero-velocity innovation $r_{k}=-H{\hat{x}}_{k}^{-}$ and evaluate the two mode likelihoods $\ell_{k}^{\left(1\right)}$ and $\ell_{k}^{\left(0\right)}$;4:Update the posterior-contact probability $\pi_{k}^{+}$ using Bayes’ rule;5:Compute the precision scale $\lambda_{k}=\pi_{k}^{+}+\left(1-\pi_{k}^{+}\right)/c$ and the effective covariance $R_{k}^{\mathrm{eff}}=R_{0}/\lambda_{k}$;6:Apply the standard ESKF correction using $R_{k}^{\mathrm{eff}}$.

## 4. Experimental Setup

### 4.1. Foot-Mounted VICON Benchmark

The core experiments in this paper use the VICON subset of the University of Toronto Foot-Mounted Inertial Navigation Dataset, released with the PyShoe toolkit (GitHub repository, master branch; no formal release version) [18,19]. The complete dataset includes VICON, hallway, and stair-climbing subsets collected under different motion conditions. In the present study, we use the VICON subset for the main benchmark because it provides dense motion-capture ground truth throughout each trial, making it the most suitable subset for controlled end-to-end evaluation of zero-velocity-aided inertial navigation.

The VICON subset was collected with a LORD MicroStrain 3DM-GX3-25 IMU (LORD MicroStrain Sensing Systems, Williston, VT, USA) mounted on the foot and sampled at 200 Hz. Ground-truth position was provided by a VICON motion-capture system operating over a compact 3×3 m capture area. The public VICON subset used here contains 56 short trials from a single subject, totaling approximately 1 km of motion across the dataset. The trials span walking, jogging, running, crawling, and ladder-climbing motions. Within each trial, the subject attempted to maintain a single dominant motion type with approximately constant gait frequency, which makes the subset suitable for controlled comparison across different ZUPT update rules.

**Figure 1:**
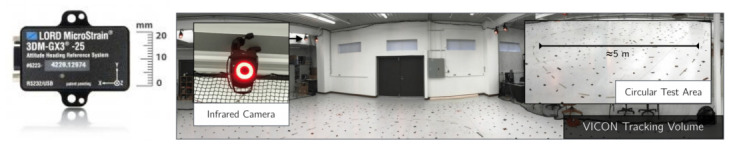
Experimental setup of the VICON benchmark used in this study. (**Left**): the LORD MicroStrain 3DM-GX3-25 foot-mounted IMU used for data collection. (**Right**): the VICON motion-capture area that provides dense ground-truth position measurements for the benchmark trials.

Figure 1 illustrates the hardware and capture setup used for this benchmark.

### 4.2. Evaluation Protocol

The main benchmark uses a two-fold development/evaluation protocol on the 56 VICON trials. To construct the folds, all trials were first sorted by the timestamps in their file names, and two complementary partitions were then generated by alternating trials in the sorted list. In Fold A, the odd-indexed trials were used for development and the even-indexed trials were used for evaluation; in Fold B, the assignment was reversed. Thus, each fold contained 28 development trials and 28 evaluation trials. The reported aggregate results were obtained by combining the two held-out evaluation folds.

The public dataset description states that the VICON subset includes walking, jogging, running, crawling, and ladder-climbing motions, and that each trial was performed with a single dominant motion type and an approximately constant gait frequency as far as possible. However, the released dataset does not provide an official trial-level mapping from trial IDs to motion types. Therefore, we do not report exact motion-type counts within each fold. Instead, we note that both folds contain trials from all recording dates, which avoids assigning an entire recording session to a single fold.

For each fold, the hard-ZUPT detector threshold is first selected on the development fold. Method-specific parameters are then selected on that same development fold and frozen before evaluation on the held-out evaluation fold.

A common operating-point policy is used throughout the main comparison. For every method, operating-point selection is performed on the development fold using mean 2D average root-mean-square error (ARMSE) as the selection objective over a predeclared parameter grid. This keeps the evaluation aligned across all compared update rules while ensuring that the evaluation fold remains held out during parameter selection. Detailed search ranges and fold-specific selected parameters are reported in the appendix.

### 4.3. Compared Methods

The main comparison includes five update rules:

- Hard-ZUPT: The classical detector-triggered baseline, in which a SHOE decision $T_{k}<G$ activates a fixed-covariance pseudo-observation with $R_{k}=R_{0}$ [9].
- Robust soft-ZUPT: The same SHOE-triggered candidate update, but with innovation-aware Student-*t* covariance scaling [31]. A Student-*t* model is a heavy-tailed alternative to a Gaussian model and is commonly used to reduce the influence of outlier-like residuals; here it serves as a robust residual-weighting rule in which larger normalized innovations are assigned larger covariance scales, reducing the Kalman gain associated with the zero-velocity pseudo-observation. Let $d_{k}^{2}=r_{k}^{⊤}S_{k}^{-1}r_{k}$, let ν denote the degrees of freedom, and let m=3 be the dimension of the zero-velocity pseudo-observation. The applied covariance scale is
  (19) $$\rho_{k}^{\mathrm{rob}}=\max\;{\left(\left(\nu +m\right)/\left(\nu +d_{k}^{2}\right),1/c_{\max}\right)}^{-1},$$
  so that $R_{k}=\rho_{k}^{\mathrm{rob}}R_{0}$.
- Contact soft-ZUPT: the detector statistic is first converted into a soft contact prior $\pi_{k}^{-}$ by the sigmoid-plus-Markov model of Section 3.4. The method then applies a contact-only covariance scale
  (20) $$R_{k}=\rho_{k}^{\mathrm{con}}R_{0},\;\rho_{k}^{\mathrm{con}}=\mathrm{clip}{\left(\pi_{k}^{-},1/c_{\max},1\right)}^{-1},$$
  without innovation-conditioned posterior correction.
- Posterior-contact soft-ZUPT: the proposed method, which starts from the same detector-derived contact prior, updates that belief using the innovation likelihood, and then applies the posterior-contact update rule of Section 3.5 and Section 3.6.
- FIBA-like adaptive covariance: a direct adaptive-covariance baseline [15] that maps the continuous SHOE instability statistic into a covariance scale according to
  (21) $$\log\rho_{k}^{\mathrm{fiba}}=2\left[\log\left(\sigma_{\mathrm{ref}}/\sigma_{\mathrm{vel}}\right)+\gamma \log\left(T_{k}/T_{\mathrm{ref}}\right)\right],$$
  followed by clipping to a prescribed range and applying $R_{k}=\rho_{k}^{\mathrm{fiba}}R_{0}$. The comparison retains the covariance-mapping form while using the same SHOE statistic and single-ESKF propagation framework as the other methods.

### 4.4. Metrics

The primary end-to-end metric is the 2D trajectory error. For a trial with aligned estimated and reference planar trajectories ${\hat{p}}_{t}^{xy}$ and $p_{t}^{xy}$, the reported trial error is the average per-sample planar root-mean-square discrepancy

(22) $$e_{i}=\frac{1}{N_{i}}\sum_{t=1}^{N_{i}}\sqrt{\frac{{\left({\hat{x}}_{i,t}-x_{i,t}\right)}^{2}+{\left({\hat{y}}_{i,t}-y_{i,t}\right)}^{2}}{2}}.$$

The 2D ARMSE quantity is used as the primary benchmark endpoint throughout the paper. We keep the planar metric as the main endpoint because planar alignment is more reliable than vertical alignment in the VICON benchmark used here. A supplementary 3D sanity check is therefore reported only in Appendix D.

The main table reports the mean, median, p90, p95, and conditional value at risk at the 90% level (CVaR@90) over the 56 trial-level values ${\left\{e_{i}\right\}}_{i=1}^{56}$. Here mean and median summarize central or typical performance, while p90 and p95 denote upper percentiles of the trial-level error distribution. CVaR@90 is defined as

(23) $${\mathrm{CVaR}}_{0.9}\left(e\right)=E\;\left[e_{i}|e_{i}\geq Q_{0.9}\left(e\right)\right],$$

that is, the average error of the worst 10% trials. In this paper, we use CVaR@90 to provide an upper-tail error summary. Unlike p90 or p95, which describe a single percentile point, CVaR@90 averages all trials beyond the 90th percentile and therefore summarizes the average severity of the difficult tail cases. It complements the mean and median summaries of typical performance and the p90/p95 summaries of high-percentile behavior. A lower CVaR@90 indicates that a method keeps errors more controlled on the most difficult trials, so it is used here to support the assessment of difficult-trial robustness and failure suppression.

## 5. Results

### 5.1. Main Results on 56 VICON Trials

Table 1 summarizes the main comparison under the fixed mean-selected protocol used in the main table.

Table 1 shows a split performance pattern across the compared update rules. FIBA-like adaptive covariance achieves the lowest mean and median error, reaching 0.168 m and 0.088 m, respectively. Posterior-contact soft-ZUPT instead achieves the lowest upper-tail metrics, with p90 = 0.400 m, p95 = 0.588 m, and CVaR@90 = 0.593 m. Robust soft-ZUPT remains highly competitive, especially on average-case metrics, while the contact-only variant is clearly weaker in the upper tail.

This pattern is consistent with the update mechanisms of the compared methods. FIBA-like adaptive covariance continuously modulates the pseudo-observation strength from the detector statistic, which appears to benefit the more regular trials that dominate mean and median performance. Posterior-contact soft-ZUPT, by contrast, combines the detector-derived prior with an innovation-conditioned posterior correction, so that suspicious candidate updates can be weakened more aggressively when the current residual is inconsistent with stable contact. The resulting behavior is less advantageous on every nominal trial, but it is more effective at suppressing rare overconfident updates, which is reflected in the stronger p90, p95, and CVaR@90 values. The contact-only ablation suggests that the detector-derived prior alone is not sufficient: without the innovation-conditioned posterior correction, tail behavior deteriorates markedly.

### 5.2. Trial-Level Pairwise Comparison

**Figure 2:**
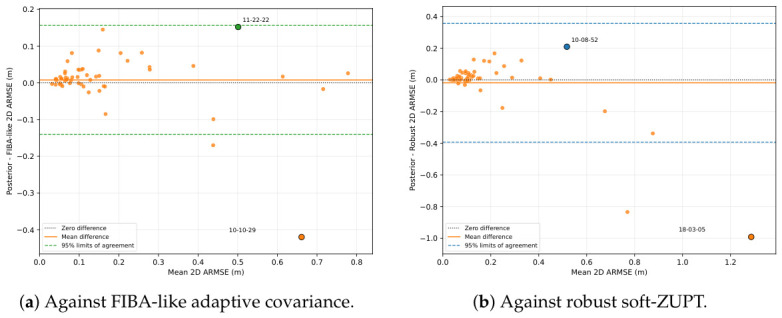
Bland–Altman trial-level comparison between posterior-contact soft-ZUPT and its two strongest comparators on the 56 VICON trials. The x-axis shows the mean 2D ARMSE of the two compared methods, and the y-axis shows posterior-contact error minus comparator error; negative differences favor posterior contact. Orange markers denote ordinary trials, while the colored markers with black outlines denote the extreme paired-difference cases highlighted by their trial labels. Both axes are expressed in meters (m).

Figure 2a,b show Bland–Altman plots comparing posterior-contact soft-ZUPT with its two strongest comparators at the trial level. For each trial, the x-axis gives the average 2D ARMSE of the two compared methods, and the y-axis gives the paired difference, defined as posterior contact minus the comparator. Therefore, negative values indicate trials on which posterior contact has lower error, whereas positive values indicate trials on which the comparator has lower error. The horizontal solid line marks the mean difference, and the dashed lines mark the 95% limits of agreement. Against FIBA-like adaptive covariance, posterior contact yields lower error on 19 of the 56 trials, while FIBA-like is lower on 37. Against robust soft-ZUPT, posterior contact is lower on 14 trials, while robust is lower on 41, with one near-tie.

As illustrated in Figure 2, most low-error trials cluster near zero difference, indicating small pairwise differences on easier trials. In contrast, the largest negative differences appear at larger average-error values, meaning that posterior contact produces its largest improvements on the more difficult trials. For example, posterior contact improves over FIBA-like by 0.420 m on trial 2018-02-22-10-10-29, whereas its largest loss to FIBA-like is 0.152 m on 2017-11-27-11-22-22. In both pairwise comparisons, the points outside the lower 95% limit of agreement occur in the negative direction, while no points exceed the upper limit. Overall, this experiment shows that posterior-contact soft-ZUPT mainly improves performance by mitigating a small number of difficult high-error cases.

### 5.3. Tail-Risk Analysis

**Figure 3:**
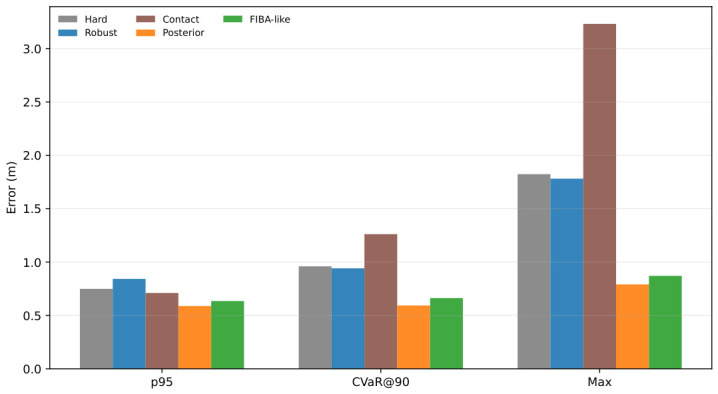
Tail-risk comparison across the selected main-table operating points, reporting p95, CVaR@90, and maximum trial error. All values are in meters (m).

Inspection of Table 1 shows that posterior-contact soft-ZUPT is strongest on the upper-tail summaries, achieving the lowest p95 and CVaR@90 among the compared methods. This pattern suggests that its main advantage may lie in suppressing rare but damaging failures rather than in uniformly improving average-case performance. To examine this point more directly, Figure 3 isolates the upper-tail metrics from the main table and augments them with the maximum trial error.

Figure 3 confirms that posterior contact achieves the lowest values on all three upper-tail summaries, reaching p95 = 0.588 m, CVaR@90 = 0.593 m, and max = 0.791 m. In comparison, FIBA-like reaches p95 = 0.635 m, CVaR@90 = 0.663 m, and max = 0.871 m, while robust soft-ZUPT reaches p95 = 0.843 m, CVaR@90 = 0.942 m, and max = 1.782 m. The contact-only ablation is notably weaker in the upper tail, with CVaR@90 = 1.262 m and max = 3.232 m.

This upper-tail advantage is consistent with the posterior-contact update mechanism. Because the method combines detector-derived contact confidence with an innovation-conditioned posterior correction, it can weaken suspicious candidate updates more aggressively when the residual is incompatible with stable contact. The resulting behavior is most visible not on the typical trials that dominate mean and median performance, but on the small number of difficult trials that determine p95, CVaR@90, and maximum error. These results therefore indicate that the principal benefit of posterior contact lies in difficult-trial mitigation and failure suppression.

### 5.4. Interpretability and Failure Analysis

The trial-level analysis reveals both posterior-favorable and FIBA-favorable cases. Figure 4, Figure 5, Figure 6 and Figure 7 show a representative posterior-favorable trial, while Figure 8, Figure 9, Figure 10 and Figure 11 show a representative FIBA-favorable trial.

**Figure 4:**
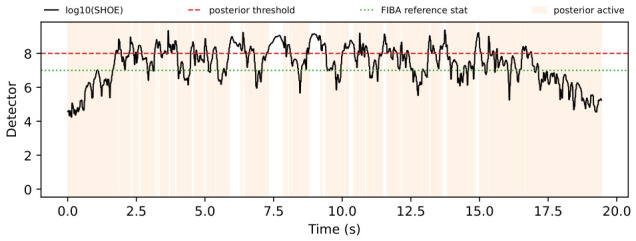
Posterior-favorable trial 2018-02-22-10-10-29: detector statistic and active posterior intervals.

**Figure 5:**
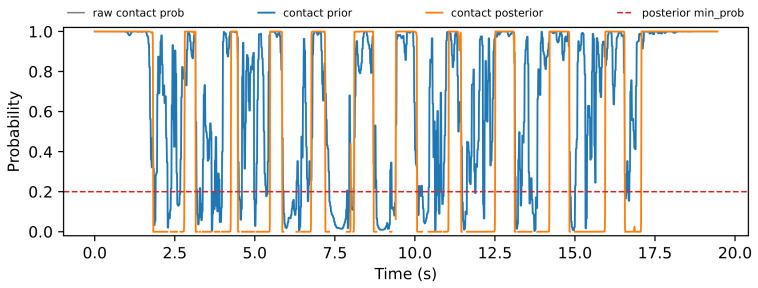
Posterior-favorable trial 2018-02-22-10-10-29: detector confidence, contact prior, and contact posterior.

**Figure 6:**
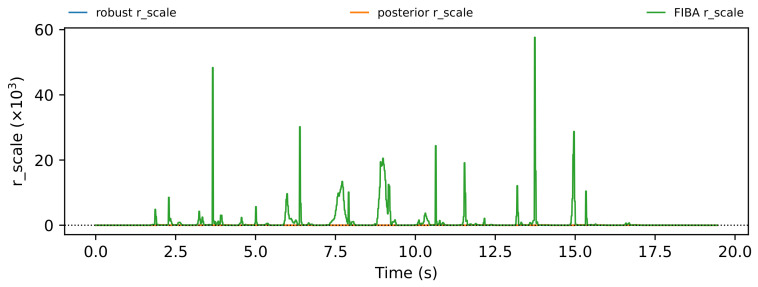
Posterior-favorable trial 2018-02-22-10-10-29: applied covariance scales for robust, posterior-contact, and FIBA-like updates.

**Figure 7:**
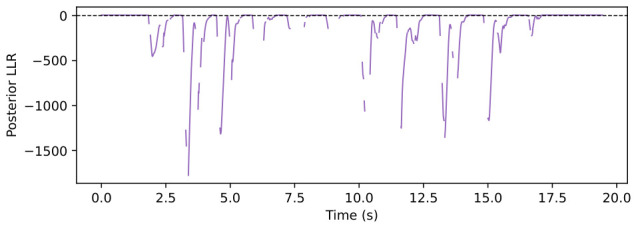
Posterior-favorable trial 2018-02-22-10-10-29: posterior log-likelihood ratio.

**Figure 8:**
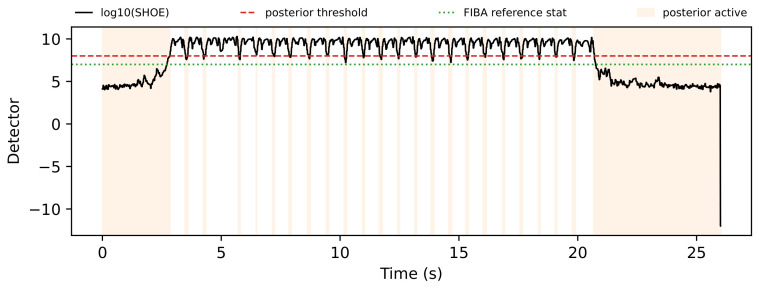
FIBA-favorable trial 2017-11-27-11-22-22: detector statistic and active posterior intervals.

**Figure 9:**
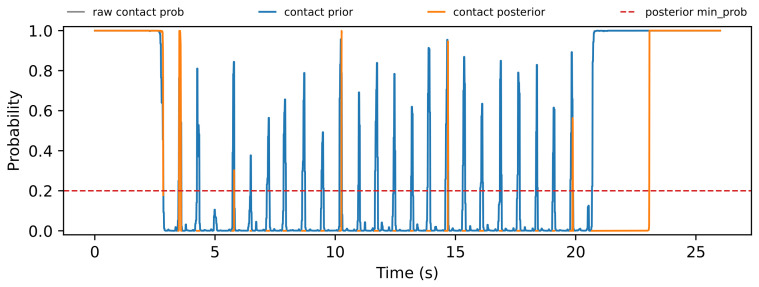
FIBA-favorable trial 2017-11-27-11-22-22: detector confidence, contact prior, and contact posterior.

**Figure 10:**
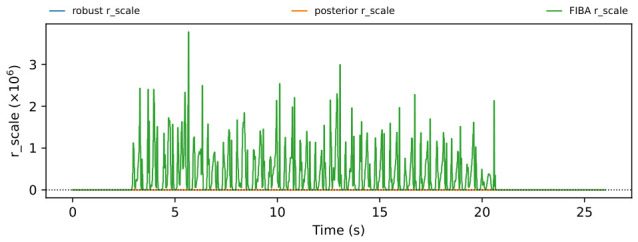
FIBA-favorable trial 2017-11-27-11-22-22: applied covariance scales for robust, posterior-contact, and FIBA-like updates.

**Figure 11:**
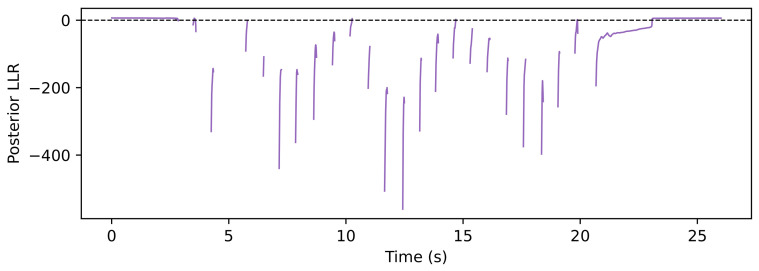
FIBA-favorable trial 2017-11-27-11-22-22: posterior log-likelihood ratio.

Figure 4, Figure 5, Figure 6, Figure 7, Figure 8, Figure 9, Figure 10 and Figure 11 provide time-aligned diagnostic views of these two trials. The detector-statistic panels show when the inertial signal is compatible with a zero-velocity/contact hypothesis; the shaded orange intervals mark the posterior-contact update intervals selected by the algorithm. These shaded intervals are not manual foot-strike or stance-phase annotations, because the public VICON benchmark provides position ground truth but not trial-level gait-event labels. The probability panels show how detector evidence is converted from raw contact confidence to a smoothed prior and then to an innovation-conditioned posterior. The covariance-scale panels show how strongly each method trusts the zero-velocity update: values near one indicate a strong update, whereas larger values weaken the update. The posterior-LLR panels show whether the innovation supports or rejects stable contact, with negative values indicating evidence against applying a confident contact update. In an ideal behavior, confident stance-like intervals would retain high contact probability and low covariance scale, while suspicious transition intervals such as heel-strike, toe-off, or unstable contact would reduce the posterior probability and increase the covariance scale.

Figure 4, Figure 5, Figure 6 and Figure 7 show that posterior contact helps when the detector alone is uncertain and the innovation indicates that a confident zero-velocity update would be unsafe. In Figure 4, the SHOE statistic repeatedly moves through ambiguous intervals near the posterior threshold, indicating that the zero-velocity hypothesis is not cleanly separated from non-contact. In Figure 5, these ambiguous intervals are followed by sharp drops from the contact prior to the posterior-contact probability. The posterior LLR in Figure 7 is strongly negative over the same intervals, showing that the innovation is actively rejecting stable-contact updates rather than merely inheriting detector skepticism. This posterior correction is then converted into covariance modulation in Figure 6: the posterior effective *r*-scale rises sharply and remains elevated over the suspicious segments, while the FIBA scale follows a smoother continuous response. Together, these patterns indicate that posterior contact recognizes risky candidate contact intervals and deliberately weakens those updates. This difference carries through to the end-to-end trial error. In this case, suppressing those locally dangerous updates reduces the 2D trajectory error from 0.871 m for FIBA-like adaptive covariance to 0.451 m for posterior-contact soft-ZUPT.

Figure 8, Figure 9, Figure 10 and Figure 11 show a FIBA-favorable regime in which the detector statistic evolves in a more regular and repetitive pattern, without the same sequence of strongly suspicious local intervals. The posterior probability in Figure 9 still reacts to the detector and innovation, but its thresholded behavior is comparatively coarse. In Figure 10, FIBA-like adaptation tracks the oscillatory instability pattern more smoothly, whereas posterior contact alternates more abruptly between strong and weak correction. As a result, the smoother continuous modulation of FIBA-like yields the lower 2D trajectory error, with 0.425 m for FIBA-like and 0.577 m for posterior contact. Posterior contact remains competitive with the hard and robust baselines.

Taken together, these two cases show that the difference between posterior contact and FIBA-like is not simply one of average strength, but of update mechanism. Posterior contact is most advantageous when detector ambiguity is followed by innovation evidence that strongly contradicts stable contact, because the posterior correction can aggressively suppress misleading updates. FIBA-like is strongest when the motion pattern is sufficiently regular so that a continuous instability-to-covariance mapping already captures the local reliability structure without the need for thresholded posterior intervention. Additional favorable and unfavorable cases in the appendix follow the same qualitative pattern.

### 5.5. Sensitivity and Regime Analysis

**Figure 12:**
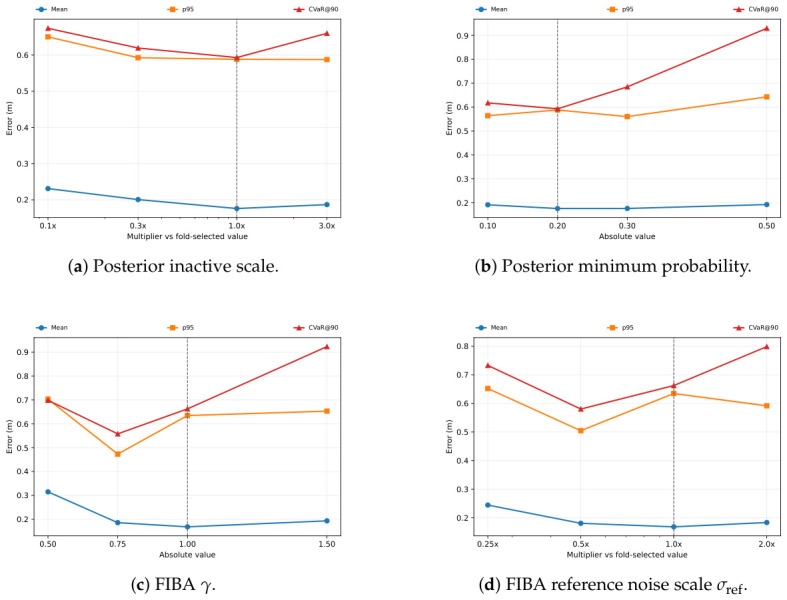
One-factor-at-a-time sensitivity analysis around the fold-selected posterior-contact and FIBA-like configurations. Each panel varies one parameter while holding the other selected parameters fixed. The plotted error metrics are in meters (m).

We next examine how the selected posterior-contact and FIBA-like configurations behave under one-factor-at-a-time parameter perturbations. Figure 12 summarizes the resulting mean, p95, and CVaR@90 curves, with the posterior-contact sweeps shown in Figure 12a,b, and the FIBA-like sweeps shown in Figure 12c,d. In this paper, a sensitivity sweep means that one parameter is varied across several candidate values while the other selected parameters are kept fixed. Thus, each panel shows how the error changes when a single design choice is made more or less conservative. The x-axis gives the parameter being varied, and the y-axis reports the resulting error metric; lower curves indicate better performance, while flatter curves indicate lower sensitivity to that parameter. Based on this reading, posterior contact shows a comparatively broad operating basin around inactive_scale = 100 and min_prob in the range 0.2–0.3. Shrinking inactive_scale to 10 raises the mean from 0.176 m to 0.231 m and the p95 from 0.588 m to 0.650 m, while increasing it to 300 keeps p95 nearly unchanged at 0.587 m but worsens CVaR@90 from 0.593 m to 0.660 m and increases the maximum error from 0.791 m to 0.962 m. Likewise, setting min_prob too aggressively at 0.5 raises CVaR@90 to 0.930 m and the maximum error to 1.804 m.

The FIBA-like sweeps show a different pattern. The fold-selected configuration remains the strongest average-case choice, with the lowest mean at gamma = 1.0 and the nominal sigma_ref values (combined mean 0.168 m). However, more conservative settings improve the upper-tail metrics: gamma = 0.75 reduces p95 from 0.635 m to 0.473 m and CVaR@90 from 0.663 m to 0.558 m, while halving sigma_ref reduces p95 to 0.505 m and CVaR@90 to 0.581 m. This indicates that FIBA-like has more headroom for tail-oriented retuning, whereas the selected posterior-contact configuration already lands at a comparatively reliability-oriented point without changing the selection objective.

**Figure 13:**
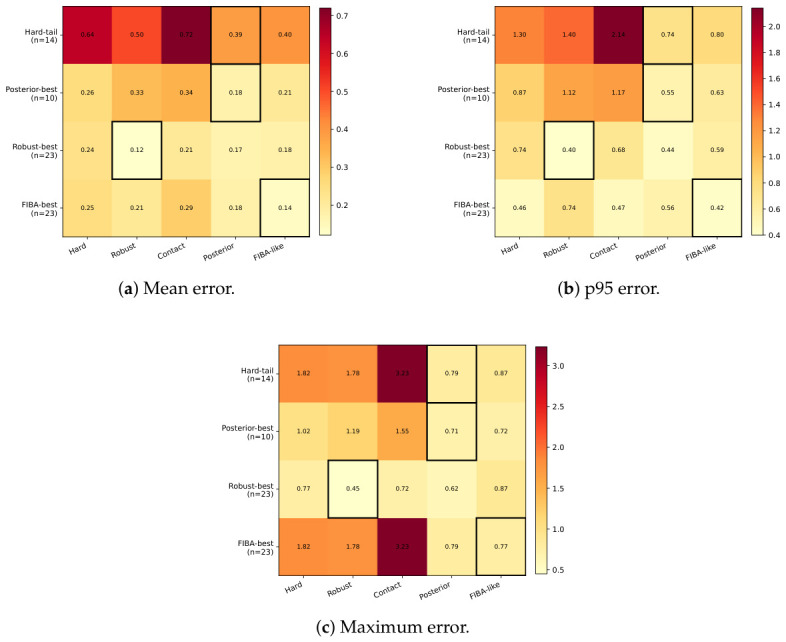
Subset-level error summaries for the main methods. Each panel compares the methods within selected trial subsets; lower values indicate better performance. Mean, p95, and maximum errors are reported in meters (m).

We also examined trial subsets to localize where the observed gains arise. Here, a trial subset means a smaller group of trials selected according to a shared property, so that performance can be inspected in a more targeted regime rather than only over all 56 trials. The most informative subset is the hard-tail regime, defined as the top quartile of hard-ZUPT errors with $e_{i}^{\mathrm{hard}}\geq 0.332$ m (n=14). This subset represents the trials where the classical hard-ZUPT baseline has the largest errors, and therefore highlights the difficult cases where failure suppression matters most. Figure 13 reports subset-level mean, p95, and maximum error for the main methods in Figure 13a–c, respectively. In these panels, lower bars indicate better performance, and the three metrics respectively summarize average error, high-percentile error, and worst-case error within the subset.

On the hard-tail subset, posterior contact is the strongest method on all three shown metrics, reaching mean = 0.387 m, p95 = 0.736 m, and max = 0.791 m, slightly ahead of FIBA-like (0.403 m, 0.802 m, 0.871 m) and well ahead of robust soft-ZUPT (0.504 m, 1.395 m, 1.782 m). In practical terms, this means that posterior contact reduces not only the average error in the difficult subset, but also the high-percentile and worst-case errors. This result further supports the view that posterior contact is most valuable on the difficult trials where failure suppression matters most.

The remaining subsets show how the other methods behave when each update rule is most naturally matched to the trial. On trials where posterior contact attains the lowest error, FIBA-like remains comparatively close, whereas robust degrades more substantially, especially on p95 and maximum error. On trials where robust is best, posterior contact remains the closest competitor and is clearly stronger than FIBA-like on the upper-tail metrics. On trials where FIBA-like is best, posterior contact again remains competitive in mean and maximum error, but FIBA-like opens a clearer advantage on p95. Taken together, these subsets suggest that posterior contact is neither broadly dominant nor brittle: it is strongest on the difficult trials emphasized by the hard-tail subset, and otherwise tends to remain near the leading method.

## 6. Discussion

The VICON results show a clear division of roles among the leading methods. FIBA-like adaptive covariance is the strongest average-case baseline, robust soft-ZUPT remains a competitive internal comparator, and posterior-contact soft-ZUPT achieves the best p90, p95, and CVaR@90 values in Table 1. The interpretation that these upper-tail gains mainly reflect difficult-trial mitigation is then supported by the pairwise trial-level comparisons, the dedicated tail-risk readout, and the hard-tail subset analysis. Taken together, these results suggest that the main value of posterior contact is not uniform trial-wise improvement, but targeted mitigation of rare overconfident update failures.

This interpretation also clarifies the methodological role of the proposed model. The contribution is not a detector replacement, but a probabilistic pseudo-observation rule that combines detector-derived contact confidence with innovation-conditioned correction inside a single ESKF update. In more regular motion regimes, continuous covariance adaptation can be more favorable on average. The sensitivity analysis shown in Figure 12 also shows that FIBA-like can improve upper-tail metrics under more conservative settings of γ or $\sigma_{\mathrm{ref}}$, although those settings move away from the mean-ARMSE-selected operating point used for the main comparison. In ambiguous contact regimes, however, the posterior-contact mechanism is able to suppress suspicious updates more aggressively once the innovation becomes inconsistent with stable zero velocity. The case studies and subset-level analyses both support this reading.

The current implementation also defines the present method boundary. In the deployed algorithm, the posterior variable primarily acts as an update-control quantity after a candidate ZUPT has been formed, rather than as a fully calibrated contact estimator or a full latent-state filter bank. This practical single-filter realization is sufficient to deliver the difficult-trial and upper-tail improvements observed here, while leaving room for future work on stronger posterior formulations and mixture-style realizations. The supplementary analyses in the appendix, including transfer, 3D sanity checks, and approximation-quality diagnostics, are broadly consistent with the same interpretation.

## 7. Conclusions

This paper presented posterior-contact soft-ZUPT as a lightweight probabilistic pseudo-observation rule for foot-mounted inertial navigation. The method combines detector-derived contact confidence with innovation-conditioned posterior correction inside a single ESKF, so that candidate ZUPTs can be weakened when the residual is inconsistent with stable contact. We evaluated the method on the 56-trial foot-mounted VICON benchmark under a two-fold development/evaluation protocol against hard-ZUPT, robust soft-ZUPT, contact soft-ZUPT, and a FIBA-like adaptive-covariance baseline.

The experiments show that posterior-contact soft-ZUPT is most valuable as a failure-mitigation mechanism for difficult trials. At the selected operating point, it achieved the strongest upper-tail profile among the compared methods, including p95 = 0.588, CVaR@90 = 0.593, and maximum error = 0.791, while the hard-tail and case-study analyses further showed that these gains arise from suppressing suspicious updates when the innovation becomes inconsistent with stable contact. These results indicate that probabilistic reliability modeling provides an effective way to soften ZUPT while preserving a lightweight single-filter implementation. Future work can extend this idea toward richer latent-state formulations, less prior-gated posterior inference, and broader validation on longer and more diverse pedestrian benchmarks.

## Figures and Tables

**Table 1:** Main results on the 56-trial foot-mounted VICON benchmark. All reported error metrics are 2D ARMSE values in meters (m).

Method	Mean (m)	Median (m)	p90 (m)	p95 (m)	CVaR@90 (m)
Hard-ZUPT	0.247	0.131	0.593	0.749	0.961
Robust soft-ZUPT	0.195	0.095	0.407	0.843	0.942
Contact soft-ZUPT	0.268	0.112	0.600	0.711	1.262
Posterior-contact soft-ZUPT	0.176	0.114	0.400	0.588	0.593
FIBA-like adaptive covariance	0.168	0.088	0.457	0.635	0.663

**Figure A1:**
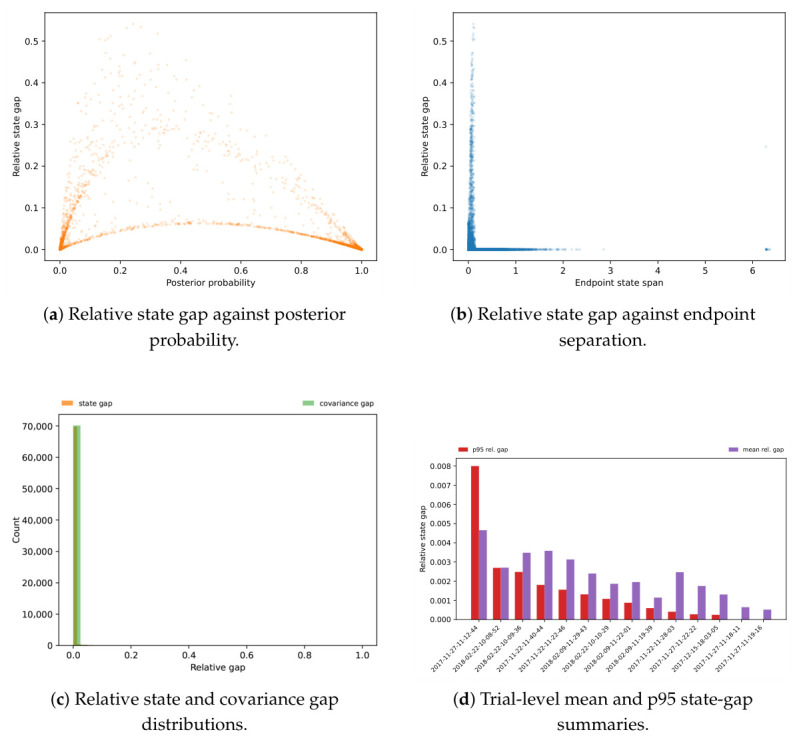
Approximation-quality overview for the single-Gaussian reduction used in posterior-contact soft-ZUPT on the 14 hard-tail VICON trials.

## Data Availability

The data presented in this study were derived from publicly available resources. The original dataset is the University of Toronto Foot-Mounted Inertial Navigation Dataset, released with the PyShoe toolkit, and is publicly available at https://starslab.ca/foot-mounted-inertial-navigation-dataset/ (accessed on 4 May 2026). Manuscript-specific processed results are available from the corresponding author on reasonable request.

## Abbreviations

The following abbreviations are used in this manuscript.

ARMSE	average root-mean-square error
CVaR	conditional value at risk
ESKF	error-state Kalman filter
FIBA	foot-instability-based adaptive covariance
IMU	inertial measurement unit
SHOE	stance hypothesis optimal estimation detector
ZUPT	zero-velocity update

## Appendix A. Selected Parameters and Search Grids

Table A1 and Table A2 report the fold-specific selected parameters and the predeclared search grids used in the main comparison.

**Table A1:** Selected fold-level parameters for the main compared methods.

Method	Fold	Selected Parameters
Hard-ZUPT	A and B	threshold = $1\times 10^{8}$
Robust soft-ZUPT	A	dof = 1, max_r_scale = 100
Robust soft-ZUPT	B	dof = 5, max_r_scale = 100
Contact soft-ZUPT	A	alpha = 4, stay_prob = 0.98, min_prob = 0.2, max_r_scale = 30
Contact soft-ZUPT	B	alpha = 4, stay_prob = 0.98, min_prob = 0.5, max_r_scale = 30
Posterior contact	A	alpha = 8, stay_prob = 0.5, min_prob = 0.2, inactive_scale = 100
Posterior contact	B	alpha = 4, stay_prob = 0.5, min_prob = 0.2, inactive_scale = 100
FIBA-like	A	reference_stat = $3\times 10^{7}$, sigma_ref = 0.05, gamma = 1.0
FIBA-like	B	reference_stat = $1\times 10^{7}$, sigma_ref = 0.01, gamma = 1.0

Table A1 shows that the selected operating points vary across folds for the adaptive methods, while the hard-ZUPT baseline uses the same selected threshold in both folds.

All operating points were selected by minimizing development-fold mean 2D ARMSE in meters over the corresponding predeclared grids.

Table A2 shows that the comparison used predeclared grids of different sizes, ranging from five hard-ZUPT thresholds to 60 FIBA-like covariance-mapping configurations.

**Table A2:** Hyperparameter search spaces for the five main compared methods.

Method	Search Space	Configs
Hard-ZUPT	$G\in \{10^{6},10^{7},3\times 10^{7},10^{8},3\times 10^{8}\}$	5
Robust soft-ZUPT	ν∈{1,3,5,10}, $c_{\max}\in \left\{10,30,100\right\}$	12
Contact soft-ZUPT	α∈{4,8}, $p_{\mathrm{stay}}\in \left\{0.5,0.98\right\}$, $\pi_{\min}\in \left\{0.2,0.5\right\}$, $c_{\max}\in \left\{30,100\right\}$	16
Posterior contact	α∈{4,8}, $p_{\mathrm{stay}}\in \left\{0.5,0.98\right\}$, $\pi_{\min}\in \left\{0.2,0.5\right\}$, $c_{\max}\in \left\{30,100\right\}$	16
FIBA-like	$T_{\mathrm{ref}}\in \left\{10^{6},10^{7},3\times 10^{7},10^{8},3\times 10^{8}\right\}$, $\sigma_{\mathrm{ref}}\in \left\{0.005,0.01,0.02,0.05\right\}$, γ∈{0.5,1.0,1.5}	60

## Appendix B. Controlled-Comparison Note on the FIBA-like Baseline

The FIBA-like baseline is designed to preserve the central idea of Jao and Shkel [15], namely continuous covariance modulation from a foot-instability statistic, within the same navigation and evaluation framework used by the other methods in this paper. Concretely, it reuses the same SHOE-derived instability statistic, the same single-ESKF navigation stack, the same development/evaluation split, and the same development-fold mean-ARMSE operating-point selection rule as the other compared methods.

Accordingly, this baseline should be understood as a controlled-comparison implementation of the covariance-mapping idea in FIBA, rather than as a full reproduction of every preprocessing, detector, and estimator detail in the original pipeline. This design keeps the surrounding detector and filter structure aligned across methods so that the comparison isolates differences in how the ZUPT covariance is scheduled.

## Appendix C. Approximation-Quality Check for the Single-Gaussian Reduction

To assess how closely the precision-matched single-Gaussian reduction tracks a local two-mode reference, we compared the deployed update against a one-step two-mode construction on the 14 hard-tail VICON trials. For each active posterior-contact step, we computed the standard deployed correction, the contact-mode endpoint update, the non-contact-mode endpoint update, and the posterior-weighted mixture moments obtained from those two endpoint corrections. The approximation gap was then measured between the deployed single-ESKF posterior and the corresponding mixture-moment reference at the same time step.

The resulting hard-tail subset contains 71,158 active posterior-contact steps. Relative to the local mixture-moment reference, the deployed reduction has mean and p95 relative state gaps of 0.0021 and 0.0009, respectively, with mean and p95 relative covariance gaps of 0.0033 and 0.0003. The trial-level summaries are similarly small: across all 14 hard-tail trials, the worst trial-level p95 relative state gap is 0.0080 and the worst trial-level p95 relative covariance gap is 0.0047.

Figure A1 helps explain why these aggregate gaps remain small. Figure A1a shows that most active steps have posterior probabilities very close to 0 or 1, so the local update is already close to a single-mode correction. Figure A1b shows that the larger approximation gaps arise mainly when the contact and non-contact endpoint updates are more widely separated. In other words, the reduction remains tight over most active steps and becomes least accurate only in the subset of steps where the two local mode-conditioned corrections disagree most strongly.

Figure A1c shows the same pattern in distributional form. Both the relative state gap and the relative covariance gap are concentrated near zero, with only a small tail of outliers. This explains why the mean and p95 summaries remain very small even though a few isolated steps exhibit visibly larger local deviations. Figure A1d shows that these deviations do not dominate at the trial level: even on the hard-tail subset, the approximation error remains localized to a small number of steps rather than spreading across entire trajectories.

The outliers are nevertheless informative. They occur most clearly when the prior still assigns substantial weight to contact, but the innovation sharply suppresses the posterior-contact probability. Those steps are precisely the cases in which the local two-mode mixture is least well represented by a single-Gaussian reduction. For the present study, the reduction is sufficiently accurate as a lightweight single-filter realization. At the same time, these outliers identify the regimes in which richer mixture-style or filter-bank formulations would be most informative in future work.

## Appendix D. VICON 3D Sanity Check

Because the VICON benchmark used here is more reliable in the horizontal plane than along the vertical axis, the main paper reports only the 2D benchmark in the core table. For completeness, Table A3 re-evaluates the already selected fold-specific operating points under the corresponding 3D ARMSE summary, without changing any thresholds or hyperparameters.

The 3D sanity check preserves the same qualitative split seen in the primary 2D benchmark. FIBA-like remains the strongest average-case method, with lower mean and median 3D error than posterior contact (0.200 m versus 0.218 m for the mean, and 0.110 m versus 0.132 m for the median). Posterior contact nevertheless retains the stronger upper-tail profile, reaching p95 = 0.576 m versus 0.721 m for FIBA-like and 0.934 m for robust soft-ZUPT, with CVaR@90 = 0.714 m versus 0.751 m and 1.212 m, respectively. The maximum 3D error remains slightly lower for FIBA-like (1.151 m versus 1.289 m). Overall, the 3D view preserves the same qualitative interpretation as the primary 2D benchmark.

**Table A3:** Supplementary 3D summary for the selected VICON operating points. All reported ARMSE values are in meters (m).

Method	Mean 2D (m)	Mean 3D (m)	Median 3D (m)	p95 3D (m)	CVaR@90 3D (m)	Max 3D (m)
Hard-ZUPT	0.247	0.260	0.176	0.695	0.969	2.106
Robust soft-ZUPT	0.195	0.244	0.103	0.934	1.212	2.303
Contact soft-ZUPT	0.268	0.306	0.190	0.728	1.236	3.279
Posterior contact	0.176	0.218	0.132	0.576	0.714	1.289
FIBA-like	0.168	0.200	0.110	0.721	0.751	1.151

## Appendix E. Cross-Dataset Hallway Transfer Pilot

To test whether the VICON-selected configurations exhibit useful behavior beyond the compact motion-capture area, we transferred the two fold-specific parameter sets directly to the hallway benchmark without any hallway-specific retuning. This dataset contains 38 hallway trials and provides sparse intermediate position ground truth rather than the dense frame-by-frame reference used in the VICON experiments. The resulting errors are therefore not numerically comparable to the main table, but they are still informative as an external transfer check on longer trajectories.

**Table A4:** Cross-dataset hallway transfer of the VICON-selected configurations. Metrics are sparse-ground-truth 2D ARMSE on 38 hallway trials and are reported in meters (m).

Transfer Config	Method	Mean (m)	Median (m)	p95 (m)	Max (m)
Fold A transfer	Hard-ZUPT	1.622	1.474	3.031	4.389
Fold A transfer	Robust soft-ZUPT	1.458	1.340	2.578	4.553
Fold A transfer	Posterior contact	1.448	1.310	2.541	4.706
Fold A transfer	FIBA-like	1.519	1.256	3.459	5.149
Fold B transfer	Hard-ZUPT	1.622	1.474	3.031	4.389
Fold B transfer	Robust soft-ZUPT	1.469	1.369	2.525	4.554
Fold B transfer	Posterior contact	1.479	1.369	2.621	4.808
Fold B transfer	FIBA-like	1.683	1.339	3.609	7.016

Table A4 shows that posterior contact transfers more stably than FIBA-like under both selected configurations. Under the Fold A transfer, posterior contact lowers the mean from 1.519 m to 1.448 m and lowers p95 from 3.459 m to 2.541 m, while under the Fold B transfer it lowers the mean from 1.683 m to 1.479 m and p95 from 3.609 m to 2.621 m. The maximum error is also reduced relative to FIBA-like in both cases.

The motion-type breakdown is particularly informative. On the 13 running trials, posterior contact is the strongest method under both transferred configurations, reaching mean = 1.038 m and 1.047 m compared with 1.658 m and 1.944 m for FIBA-like. The walking and mixed-motion subsets are more mixed, but the overall transfer pattern remains directionally consistent with the VICON benchmark: the main advantage of posterior contact continues to appear on the longer and more failure-prone trajectories rather than on the most benign cases.
